# Impact of a one-time formative OSCE on learning behavior and self-assessment in dental undergraduate education

**DOI:** 10.1186/s12909-025-08533-5

**Published:** 2026-01-09

**Authors:** Thekla J. Pfeiffer-Grötz, Friederike Basten, Anke Hollinderbäumer, Lisa Zöll, James Deschner

**Affiliations:** 1 Department of Periodontology and Operative Dentistry, University Medical Center of the Johannes Gutenberg University, Augustusplatz 2, Mainz, 55131 Germany; 2 University Medical Center, University of Mainz, Rudolf Frey Lernklinik, Mainz, Germany

**Keywords:** OSCE, Dental education, Examination form, Learning status, Learning behavior, Learning motivation

## Abstract

**Background:**

With the introduction of the new dental licensing regulations (ZApprO) in Germany, preclinical teaching time was substantially reduced, particularly affecting practical training. To support students’ learning under these conditions, a formative Objective Structured Clinical Examination (OSCE) was implemented early in the preclinical curriculum. This study aimed to evaluate the impact of an early formative OSCE on undergraduate dental students’ learning behavior, self-assessment, and exam preparation.

**Methods:**

A total of 71 undergraduate dental students (mean age 22 years) participated voluntarily in a formative OSCE in preventive dentistry during the summer semester 2022 and winter semester 2022/23. Students were randomly assigned to an intervention group (OSCE halfway through the semester) or a control group (OSCE shortly before the final exam). The OSCE included five stations developed according to the National Competence-Based Learning Objectives Catalog for Dentistry. Students completed pseudonymized questionnaires at two time points (T0: after OSCE; T1: after the final exam). The questionnaire assessed learning behavior (including strategies for dealing with difficult material and use of additional resources), self-assessment (perceived learning status and exam readiness), motivation, and exam preparation. Statistical analyses were performed using Mann–Whitney U and Wilcoxon tests.

**Results:**

Participation in the formative OSCE enabled students to better evaluate their learning status and identify individual learning needs for the final exam. Although both groups started exam preparation at similar times (T0: *p* = 0.422; T1: *p* = 0.674), the intervention group reported higher initial motivation and greater awareness of knowledge gaps after the OSCE. Differences were also observed in how students dealt with difficult material and in their use of supplementary learning resources.

**Conclusions:**

An early formative OSCE fosters undergraduate dental students’ self-assessment, reflection on learning behavior, and awareness of learning needs, thereby supporting more targeted exam preparation. However, it does not necessarily lead to an earlier start of study activities. Implementing formative OSCEs in the middle of the semester, accompanied by structured feedback, may further enhance their educational impact.

**Supplementary Information:**

The online version contains supplementary material available at 10.1186/s12909-025-08533-5.

## Introduction

Since the winter semester 2021/22, a modified dental licensing regulation (ZApprO) has been in effect in Germany. One major change is a reduction of preclinical teaching hours by about 74%, particularly affecting practical training. This creates challenges in adequately preparing dental students for clinical requirements. In dentistry, however, the acquisition of practical skills and the ability to critically reflect on one’s own competence are crucial from the very beginning of training [1].

Objective structured clinical examinations (OSCEs) have been widely used in medical and dental education since the 1970s to assess clinical competence in a structured, standardized way. They consist of a series of short stations with defined tasks, enabling students to demonstrate knowledge, practical skills, and decision-making under exam conditions. While OSCEs are often used in clinical phases, their formative use in early preclinical semesters is less common [2–4].

In this context, student success in preclinical dental education can be understood not only as exam performance, but more broadly as the ability to realistically assess one’s learning status, to develop effective learning strategies, and to prepare adequately for examinations. Formative assessments such as the OSCE may provide students with valuable feedback to support these aspects of success [5].

The aim of this study was therefore to analyze the effect of a one-time formative OSCE in the second preclinical semester on students’ learning behavior, self-assessment, and exam preparation in preventive dentistry. In addition, the study compared different time points of OSCE implementation (mid-semester vs. shortly before the final exam) in order to identify when in the semester this formative tool may be most effective.

## Materials and methods

### Participants/ target group

The preventive dentistry lecture series in the second preclinical semester at Johannes Gutenberg University Mainz served as the study setting. A total of 71 dental students participated voluntarily: 32 in summer semester 2022 and 39 in winter semester 2022/23. The average age was 22 years (range 19–28); 18 were male and 53 female. Written informed consent was obtained, and participation could be withdrawn at any time. Twenty-nine students had to be excluded due to incomplete data. The study was previously presented to the Ethics Committee Rhineland-Palatinate, which waived the requirement for a formal procedure number as the project was classified as educational quality assurance. All participants were fully informed about the study procedures and took part voluntarily. Each participant completed the formative OSCE and at least one of the questionnaires.

### Study design and groups

Students were randomly assigned to two groups. The intervention group (IG) completed a formative OSCE halfway through the semester (week 7), while the control group (CG) completed the OSCE in week 11, shortly before the final exam in preventive dentistry (week 12). This design allowed comparison of the influence of OSCE timing on learning behavior and exam preparation. All students participated in the OSCE. The aim of the study was to compare different timings of a single formative OSCE, not to compare OSCE versus no OSCE. Therefore, no group without OSCE participation was included.

### OSCE format

The formative OSCE consisted of five stations (morphology of teeth, oral hygiene, instruments, anatomy of the oral cavity, dental hard tissue defects and caries diagnosis). Each station lasted eight minutes, with a two-minute changeover period. The stations were developed according to the National Competence-Based Learning Objectives Catalog for Dentistry (NKLZ) and a standardized blueprint provided by the Rudolf Frey Learning Clinic. The focus was on the application of preclinical course content rather than clinical skills. A maximum of 25 points could be achieved per station, but scores were not used for grading. Participation was voluntary and formative, designed to provide students with an opportunity for self-assessment. As the OSCE was implemented as a formative, ungraded assessment, no performance scores were recorded for analytical purposes, and students did not receive individual results. Therefore, no correlation with final exam grades was possible.

### Data collection

Data were collected using two structured, pseudonymized questionnaires administered at two time points (T0: directly after the OSCE; T1: after the final exam). Both questionnaires consisted exclusively of closed-ended items. Most questions used 5-point Likert scales (1 = very rarely to 5 = very often or 1 = strongly agree to 5/7 = strongly disagree), while a few items included single-choice or multiple-choice response formats. No open-ended or narrative questions were included; therefore, no qualitative data analysis was required.

Items on learning strategies were based on the validated LIST questionnaire [6], while items on exam preparation, motivation, and perceived learning status were developed specifically for this study and pretested for clarity.

Student self-assessment in this study referred exclusively to students’ subjective reflection on their learning strategies, perceived learning progress, and exam readiness. Because the OSCE was administered as a purely formative assessment without grading consequences, students did not receive their OSCE results, and no comparison with faculty scoring was possible. Likewise, no comparison with final exam performance was conducted. Self-assessment therefore focused solely on learners’ internal perception of their learning status rather than on performance-based evaluation.

### Statistical analysis

Data were analyzed with SPSS Statistics (IBM, version 23). As normal distribution could not be assumed, non-parametric tests were applied. Group differences were tested with the Mann–Whitney U test, and within-group changes over time with the Wilcoxon signed-rank test. Correlations were examined using Spearman’s rank correlation. A significance level of *p* < 0.05 was applied. Missing values were handled by pairwise case exclusion.

## Results

### Questions on exam preparation

**Fig. 1:**
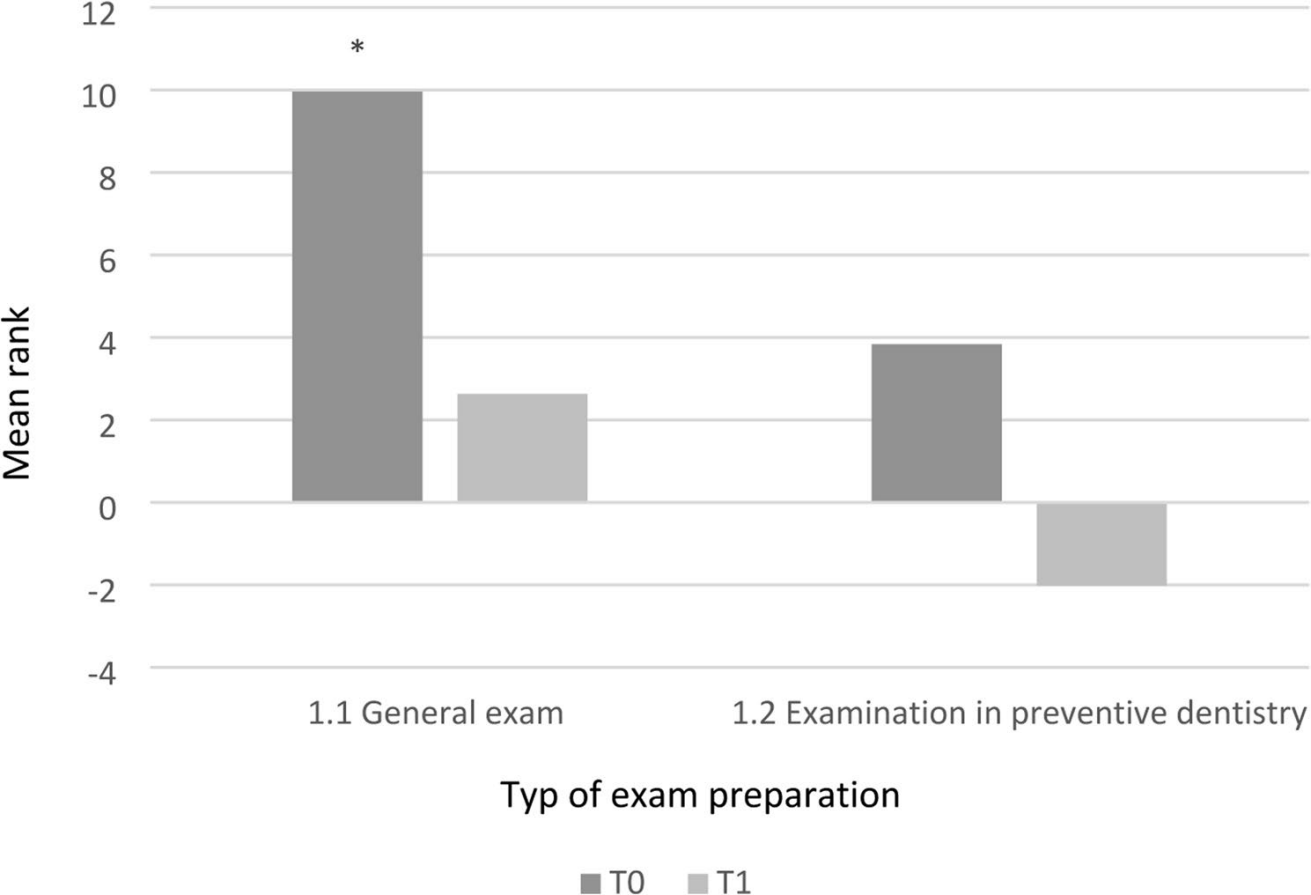
Differences in mean rank values for the timing of exam preparation between the intervention group (IG) and control group (CG) at two measurement points: T0 (after the OSCE) and T1 (after the final exam in preventive dentistry). Higher ranks indicate earlier self-reported exam preparation. Ranks represent the mean rank values derived from the Mann–Whitney U test. (*p < 0.05)

Participation in a formative OSCE halfway through the semester (intervention group, IG) led to a more realistic self-assessment of when to start preparing for the final exam in preventive dentistry compared with the control group (CG). However, no significant differences were found between the groups in the actual timing of exam preparation (*p* = 0.067) (Fig. 1).

**Fig. 2:**
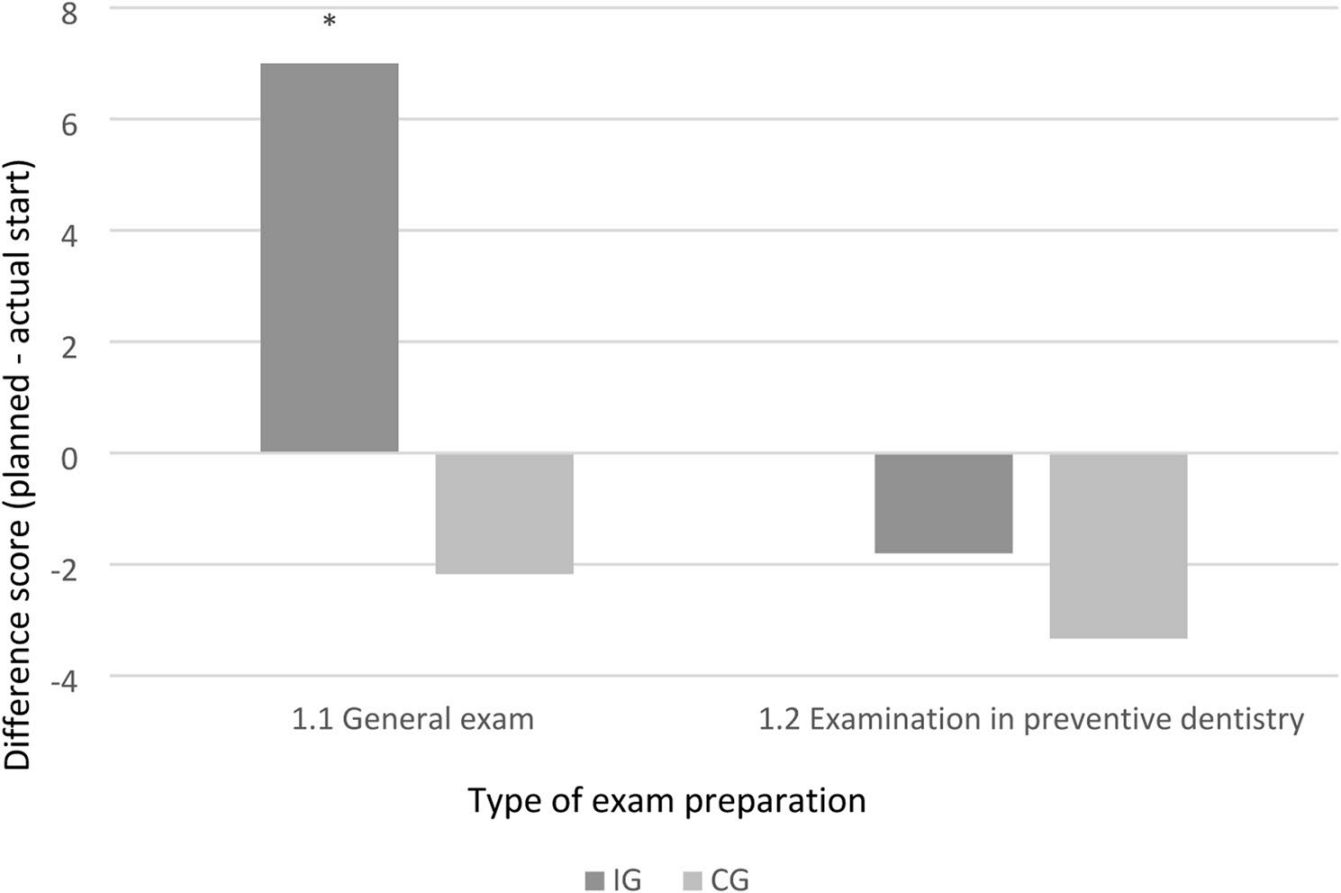
Change in the reported start of exam preparation, representing the difference between the planned and actual start, in the intervention group (IG; OSCE in week 7) and the control group (CG; OSCE in week 11). Negative values indicate a later start than originally planned. Higher positive values represent an earlier start than planned. (*p* < 0.05)

At T0 (directly after the OSCE), IG students reported an earlier planned start of learning than CG students, but this effect was not confirmed at T1 (after the final exam). Within-group analyses showed that students in both groups tended to postpone the actual start of learning compared with their original plan. In particular, IG students demonstrated a shift toward later exam preparation (*p* = 0.001) (Fig. 2). Suggesting that their initially higher motivation after the OSCE (T0) was not sustained throughout the semester.

### Questions on motivation to participate

**Fig. 3:**
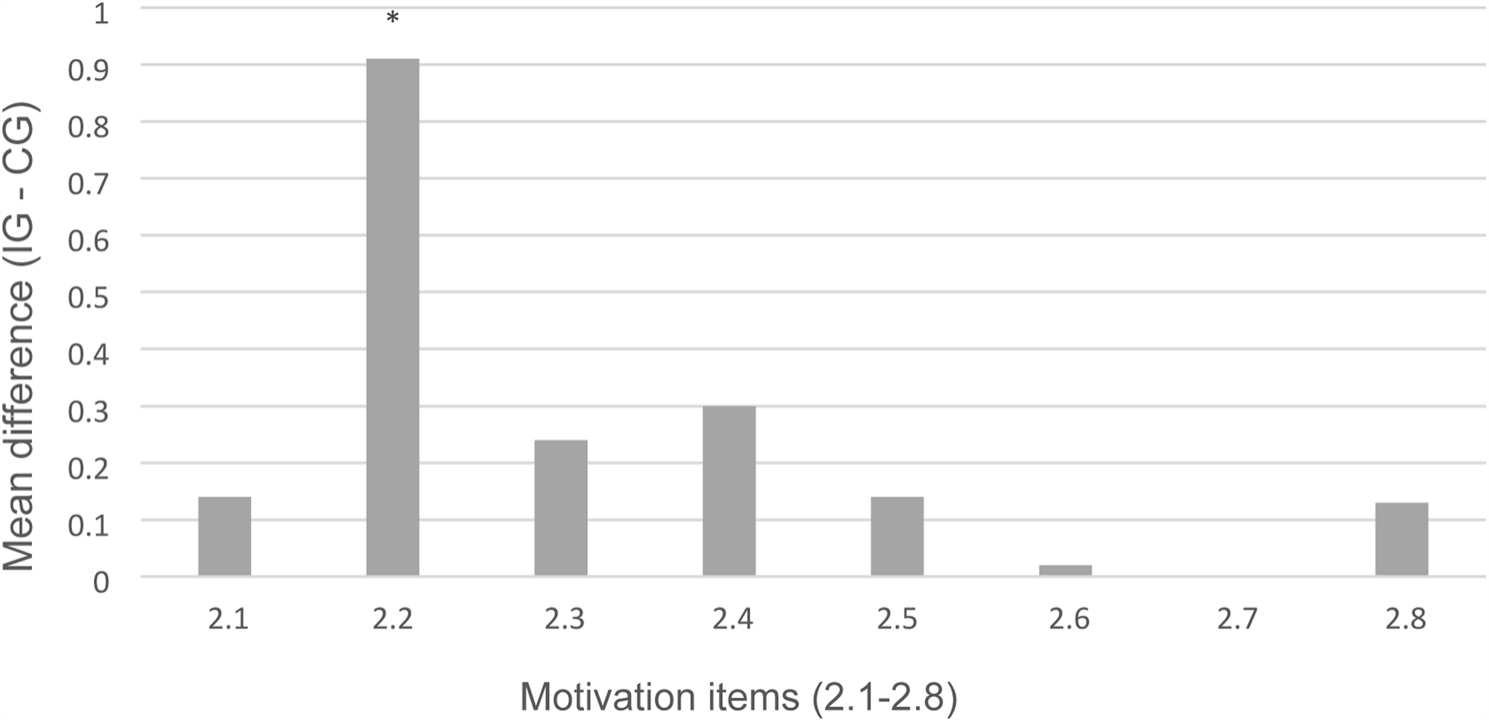
Differences between the intervention group (IG) and control group (CG) in mean scores for motivation-related items (Likert scale, 1 = strongly agree to 7 = strongly disagree) directly after OSCE participation. Positive values indicate higher mean scores in IG compared to CG. The item “The OSCE format is exciting” (item 2.2) showed a statistically significant difference between groups. (*p* < 0.05)

Overall, motivation to participate in the OSCE did not differ significantly between IG and CG. Most students agreed that the OSCE was useful for exam preparation and helped them identify relevant content. At item level, a significant difference was observed in how exciting students found the OSCE format (*p* = 0.002): IG students rated it as more engaging than CG students (Fig. 3). This may be related to the shorter interval between briefing and examination for IG compared to CG.

### Questions on the general learning strategy for exams

**Fig. 4:**
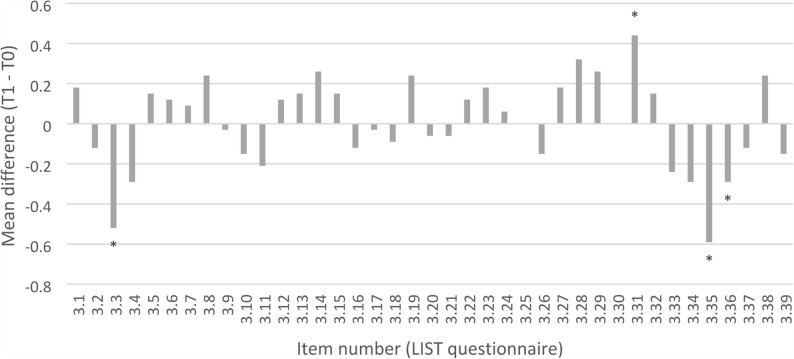
Changes in learning strategies from T0 (after the OSCE) to T1 (after the final exam) in the intervention group (IG). Values represent mean differences (T1 – T0) for items from the LIST questionnaire (Likert scale, 1 = very rarely to 5 = very often). Positive values indicate an increase in the reported behavior over time, while negative values indicate a decrease. (*p* < 0.05)

**Fig. 5:**
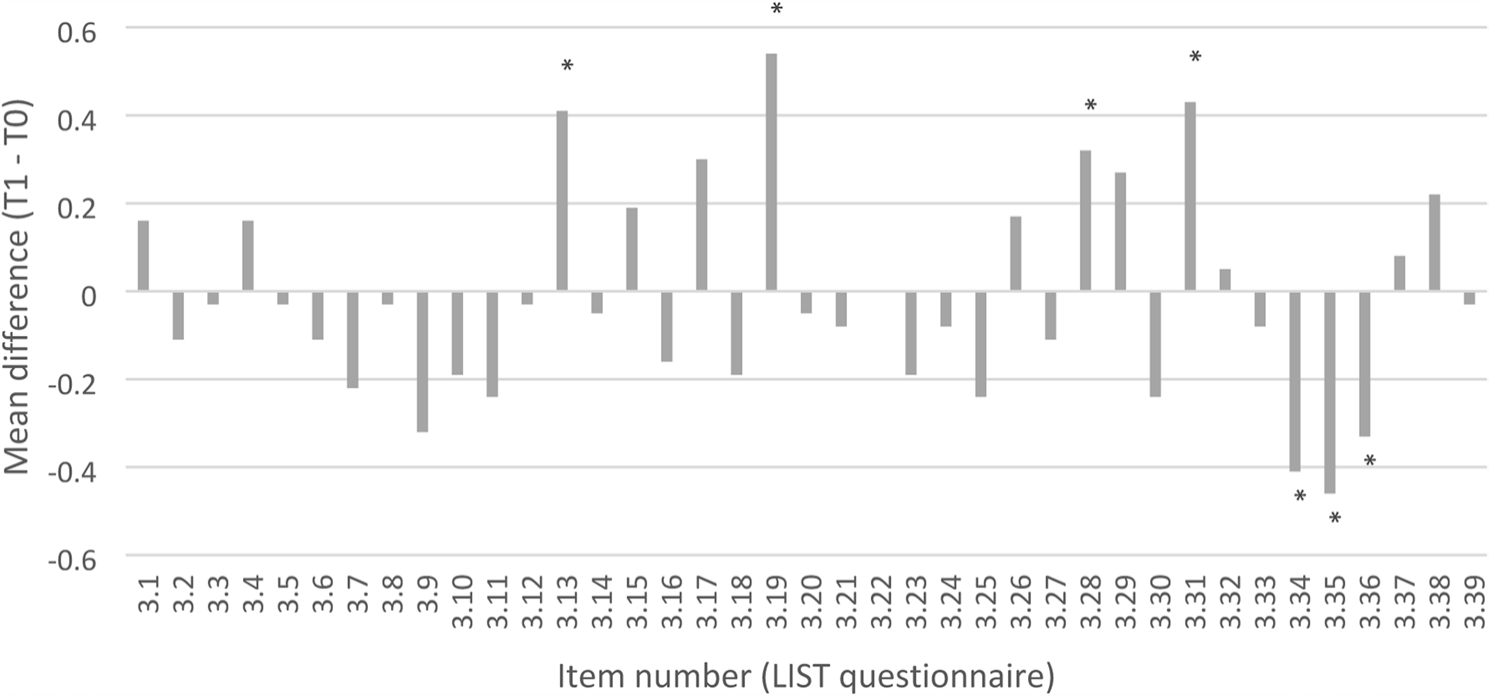
Changes in learning strategies from T0 (after the OSCE) to T1 (after the final exam) in the control group (CG). Values represent mean differences (T1 – T0) for items from the LIST questionnaire (Likert scale, 1 = very rarely to 5 = very often). Positive values indicate an increase in the reported behavior over time, while negative values indicate a decrease. (*p* < 0.05)

Analysis of general learning strategies showed no overall differences between groups or across time points. However, some item-level effects were observed: IG students were less likely to give up when faced with difficult material (*p* = 0.029), indicating greater persistence (Fig. 4). In both groups, the use of additional literature and external resources (e.g., textbooks, supplementary materials) decreased significantly from T0 to T1, while peer learning (working with fellow students) increased toward the final exam (Fig. 5).

**Fig. 6:**
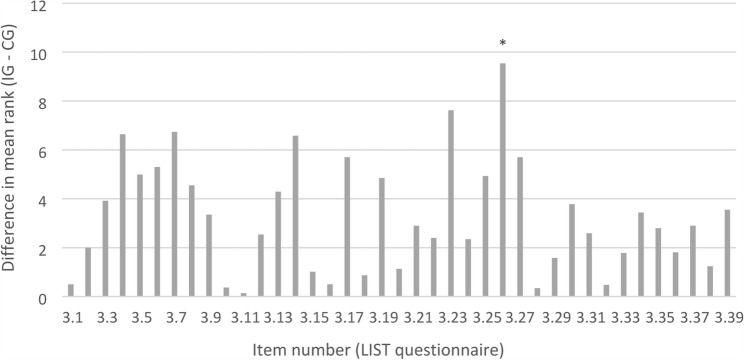
Comparison of learning strategies between the intervention group (IG) and control group (CG) at T0 and T1. Values represent differences in mean ranks (IG – CG) derived from Mann–Whitney U tests for items of the LIST questionnaire (Likert scale, 1 = very rarely to 5 = very often). Positive values indicate higher ranks in IG, and negative values indicate higher ranks in CG. (*p* < 0.05)

These findings suggest that participation in a formative OSCE influenced specific aspects of students learning strategies, particularly persistence and the selection of learning resources, but did not lead to substantial changes in overall learning behavior (Fig. 6).

## Discussion

### Overview of main findings

This study investigated the effect of a one-time formative OSCE on learning behavior, motivation, and exam preparation in undergraduate dental education. The main finding is that the timing of the OSCE influenced students’ self-assessment and certain aspects of learning behavior but did not consistently lead to an earlier start of exam preparation. Participation in the OSCE halfway through the semester helped students develop a more realistic understanding of their learning status and preparation needs, yet the intervention group tended to postpone actual exam preparation compared with their initial plans. This suggests that initial motivation after the OSCE was not sustained until the final exam. Specifically, students in the intervention group showed higher initial motivation and greater awareness of knowledge gaps after the formative OSCE, although this did not translate into an earlier actual start of exam preparation.

### Self-assessment and reflection

Our findings demonstrate that students who participated in the OSCE earlier in the semester reported clearer insight into their learning needs and higher awareness of knowledge gaps. Similar outcomes have been reported by Curtis et al. [7], who found that self-assessment exercises enhance students’ awareness of their learning progress and deficiencies. The results also align with previous work by Duerson et al. [8] and Khan et al. [9], emphasizing that formative OSCEs provide an opportunity for feedback and reflection on practical competence rather than simple knowledge testing. From a broader perspective, Beard et al. [10] demonstrated in surgical training that structured formative assessments can serve as reliable tools to evaluate skills development, supporting our interpretation that such approaches are transferable to dental education.

### Motivation and perceived usefulness

Both groups reported high motivation to participate in the OSCE, which can be attributed to its novelty and the opportunity to practice exam-relevant skills. Students in the intervention group rated the OSCE as more engaging than those in the control group, likely due to its proximity to preparatory sessions. This significant item-level difference highlights that OSCE timing can influence students’ perceived engagement with the assessment format. This observation supports findings by Schoonheim-Klein et al. [11], who reported that the perceived fairness and organization of OSCEs contribute to student engagement. However, our results also show that motivation declined over time, consistent with longitudinal research indicating that initial enthusiasm after formative assessments often diminishes without continued reinforcement [12, 13]. Formative assessments may therefore serve as short-term motivators but require integration with continuous feedback to maintain learning momentum [14].

### Learning behavior and exam preparation

While general learning strategies did not differ significantly between groups, item-level analyses revealed differences in persistence and resource management. The intervention group showed greater persistence when dealing with difficult material, whereas both groups reduced their use of external literature and online resources as the semester progressed. Instead, peer learning increased toward the final exam, reflecting the natural adaptation of study behavior observed in other student populations [15, 16]. This shift from individual to collaborative learning strategies as the exam approached corresponds with theoretical models of adaptive learning behavior, which describe an increasing reliance on peer-supported learning under rising assessment pressure. These findings correspond with the theoretical models of learning motivation proposed by Schiefele et al. [12], suggesting that internal motivational factors and learning strategies interact dynamically over time.

In the context of dental education, previous studies have shown that OSCE performance correlates with theoretical exam results, but not necessarily with broader learning habits [17]. This may explain why our participants’ exam preparation timing remained largely unchanged despite increased self-awareness. Formative assessments appear to improve reflection rather than directly alter study schedules. Because the OSCE was used solely as a formative exercise without scoring, no performance correlation with the summative final exam could be conducted.

### Educational implications

The present findings reinforce earlier evidence that formative OSCEs can enhance learning by supporting feedback-driven reflection and encouraging self-directed learning [7, 18]. However, consistent behavioral change may require more frequent exposure. Schüler et al. [19] demonstrated that structured and qualified feedback significantly improves dental students’ clinical performance, suggesting that feedback is a key mediator of learning transfer. Likewise, Gallagher [14] emphasized that integrating feedback mechanisms into constructive alignment models strengthens the educational impact of assessments. The observed increases in students’ awareness of learning needs and their greater persistence when faced with difficult material indicate that strategically timed formative OSCEs can meaningfully support the development of self-directed learning behaviors. Aligning OSCE timing with curricular milestones may also enhance effectiveness. Conducting formative OSCEs in the second half of the semester, several weeks before the final exam, could maximize their influence on exam preparation and sustained motivation. Similar timing considerations have been proposed in the context of medical and surgical education [10, 17].

### Strengths and limitations

#### Strengths

A particular strength of this study is its focus on an early formative OSCE within the preclinical phase, providing authentic insights into how such assessments influence students’ learning behavior and motivation. The assessment format was well accepted and perceived as useful and motivating, especially when implemented several weeks before the final exam. These aspects underline the practical relevance of the study within real curricular conditions.

#### Limitations

This study has several limitations. The voluntary nature of participation may have introduced selection bias, and the relatively small sample size and gender imbalance limit the generalizability of the findings. Furthermore, the reliance on self-reported questionnaires may not fully reflect actual learning behavior [20]. The OSCE was deliberately designed as a non-graded, feedback-oriented experience; however, the lack of individualized performance feedback may have reduced its formative potential [19]. Future studies could integrate personalized feedback sessions to strengthen learning outcomes and motivation.

## Conclusion

Conducting a one-time formative OSCE early in dental education can help students identify individual learning needs, support realistic self-assessment, and influence selected aspects of learning behavior, even though it does not consistently lead to earlier exam preparation or comprehensive changes in learning strategies.

A particular strength of this study is its focus on an early formative OSCE within the preclinical phase, providing valuable insights into how such interventions affect students’ learning behavior and motivation under real curricular conditions. The format was well accepted and perceived as useful and motivating, particularly when implemented several weeks before the final exam. Its educational potential may be further enhanced when embedded repeatedly within the curriculum and combined with structured feedback and explicit learning objectives.

## Supplementary Information

Supplementary Material 1.

## Data Availability

The datasets used and analysed during the current study are available from the corresponding author on reasonable request.
