# Supplementary material for: Impact of a one-time formative OSCE on learning behavior and self-assessment in dental undergraduate education
Source: BMC Med Educ. 2026 Jan 9;26:147. doi: 10.1186/s12909-025-08533-5 (PMC12849650; doi:10.1186/s12909-025-08533-5)
Supplement: Supplementary file 1 — Supplementary Material 1. [file 12909_2025_8533_MOESM1_ESM.docx]

### Questions on exam preparation

- 1. When do you usually start studying for an exam?
  2. When will you start/have you started working on learning content with regard to exam preparation for preventive dentistry?

### Questions on motivation to participate

- 1. I hope that OSCE will help me realize what is relevant to the exam.
  2. I find the OSCE method exciting.
  3. I am not yet familiar with the OSCE format.
  4. The OSCE prepares me for the exam.
  5. I want to try everything to get better.
  6. I want to learn more.
  7. I hope that the OSCE can teach me skills that I don't learn from the lectures and the final exam.
  8. I'm afraid of the consequences if I don't take part in the OSCE.

### Questions on the general learning strategy for exams

- 1. I go through my notes and make an outline with the most important points.
  2. I compile short summaries of the main ideas from my notes, script or literature.
  3. I compile important technical terms and definitions in my own lists.
  4. I try to relate new terms or theories to terms and theories I already know.
  5. I come up with concrete examples of specific learning content.
  6. I relate what I learn to my own experiences.
  7. I ask myself whether the text I am working through is really convincing.
  8. I approach most texts critically.
  9. I critically examine what I learn.
  10. I memorize a self-made overview of the most important technical terms.
  11. I memorize rules, technical terms or formulas.
  12. I memorize the learning material using scripts or other notes as far as possible.
  13. I formulate learning objectives to which I then align my learning.
  14. I think about how I want to learn before I start learning.
  15. I do not plan my approach to learning.
  16. To identify gaps in my knowledge, I recapitulate the most important content without using my notes.
  17. I ask myself questions about the material to check whether I have understood everything.
  18. If the learning material contains questions or tests, I use these to check myself.
  19. I change my learning technique when I encounter difficulties.
  20. I change my learning plans when I realize that they cannot be implemented.
  21. I change my approach to learning when I realize that it is not successful.
  22. I find it difficult to stay on task.
  23. I am unfocused when I am learning.
  24. I am easily distracted when I am studying.
  25. When I have set myself a certain amount of studying to do, I make an effort to complete it.
  26. I don't give up, even if the material is very difficult or complex.
  27. I also study late at night and at weekends if I have to.
  28. I stick to a certain schedule when I study.
  29. I use a timetable to determine the hours I spend studying each day.
  30. I set a certain amount of time before each learning phase.
  31. I work on texts or assignments together with my fellow students.
  32. I take time to discuss the material with my fellow students.
  33. If something is not clear to me, I ask a fellow student for advice.
  34. I look for further reading if certain content is not yet completely clear to me.
  35. I gather missing information from various sources (e.g. notes, books, specialist journals).
  36. I consult additional literature if my notes are incomplete.
  37. I organize my environment in such a way that I am distracted as little as possible from my studies.
  38. I always sit in the same place to study.
  39. My workplace is designed so that I can find everything quickly.
